# T cell receptor‐based cancer immunotherapy: Emerging efficacy and pathways of resistance

**DOI:** 10.1111/imr.12772

**Published:** 2019-07-29

**Authors:** Smita S. Chandran, Christopher A. Klebanoff

**Affiliations:** ^1^ Center for Cell Engineering and Department of Medicine Memorial Sloan Kettering Cancer Center New York NY; ^2^ Parker Institute for Cancer Immunotherapy New York NY; ^3^ Weill Cornell Medical College New York NY

**Keywords:** adoptive immunotherapy, CRISPR/Cas9, genetic engineering, ImmTAC, TCR mimic

## Abstract

Adoptive cell transfer (ACT) using chimeric antigen receptor (CAR)‐modified T cells can induce durable remissions in patients with refractory B‐lymphoid cancers. By contrast, results applying CAR‐modified T cells to solid malignancies have been comparatively modest. Alternative strategies to redirect T cell specificity and cytolytic function are therefore necessary if ACT is to serve a greater role in human cancer treatments. T cell receptors (TCRs) are antigen recognition structures physiologically expressed by all T cells that have complementary, and in some cases superior, properties to CARs. Unlike CARs, TCRs confer recognition to epitopes derived from proteins residing within any subcellular compartment, including the membrane, cytoplasm and nucleus. This enables TCRs to detect a broad universe of targets, such as neoantigens, cancer germline antigens, and viral oncoproteins. Moreover, because TCRs have evolved to efficiently detect and amplify antigenic signals, these receptors respond to epitope densities many fold smaller than required for CAR‐signaling. Herein, we summarize recent clinical data demonstrating that TCR‐based immunotherapies can mediate regression of solid malignancies, including immune‐checkpoint inhibitor refractory cancers. These trials simultaneously highlight emerging mechanisms of TCR resistance. We conclude by discussing how TCR‐based immunotherapies can achieve broader dissemination through innovations in cell manufacturing and non‐viral genome integration techniques.

## 1 INTRODUCTION

Despite regulatory approval of immune checkpoint inhibitors in a diverse range of human solid malignancies,^1^ it remains the case that most patients do not benefit from current cancer immunotherapies. This is particularly true for patients with modestly mutated cancers arising from epithelial organs,^2^ collectively the leading causes of adult cancer‐related deaths.^3^ Detailed immune monitoring studies of exceptional patient responders to immunotherapy have revealed three critical variables that simultaneously must be satisfied for cancer regression to occur.^4^, ^5^, ^6^, ^7^, ^8^, ^9^, ^10^, ^11^, ^12^, ^13^, ^14^, ^15^ First, the patient must possess a repertoire of T cells capable of recognizing antigens displayed on the surface of cancer cells. Second, these same antigen‐specific T cells must possess an intrinsic capacity to expand, infiltrate a solid tumor mass, and persist. Third, the T cells must remain functional within the tumor microenvironment. Patients with common epithelial malignancies are confronted with several major obstacles that limit the ability of their T cell compartments to fulfill these requirements.

Most patients with metastatic epithelial cancers have evidence of tumor‐specific T cells both within the circulation^8^, ^12^, ^16^, ^17^ and the tumor microenvironment.^8^, ^11^, ^12^, ^17^, ^18^, ^19^ Nevertheless, the mere presence of these cells appears insufficient to enable cancer regression to commonly used immunotherapies, such as immune checkpoint inhibitors.^20^, ^21^, ^22^ The biologic basis for this discrepancy remains incompletely defined. However, one likely explanation resides in the recent discovery that most antigen‐specific T cells in these patients reside within the pool of effector memory (T_EM_)/effector memory RA (T_EMRA_) T cells.^16^ The T_EM_ and T_EMRA_ populations represent the most terminally differentiated of T cell subsets. Among all memory T cells, they possess the least capacity for sustained proliferation and are most prone to senescence and apoptosis.^23^ They are, in short, incapable of expanding to the magnitude required to induce clinically apparent tumor regression. This situation is compounded in many solid cancer patients by the routine use of cytotoxic chemotherapy and radiation therapy which serves to deplete less‐differentiated T cell subsets.^24^, ^25^, ^26^ Lastly, the microenvironment of many cancers is enriched in immunosuppressive cell subsets, including T regulatory cells, aberrantly matured myeloid cells, and immunosuppressive populations of fibroblasts.^27^

Each of these limiting variables may in principle be overcome using adoptive cell transfer (ACT). In this approach, cancer‐specific T cells are isolated and expanded outside the potentially immune‐suppressive environment of a cancer patient to therapeutic numbers before reinfusion. Cancer‐specificity can be ensured using genetic engineering to introduce an exogenous antigen receptor matching the complement of antigens expressed by a patient's cancer. Specific T cell populations with enhanced proliferative and survival potential, including the minimally differentiated T stem cell memory (T_SCM_) and T central memory (T_CM_) subsets, may be selected.^28^ Finally, because the therapeutic T cells are expanded ex vivo, the tumor‐bearing host may be preconditioned to deplete immune‐suppressive cell populations prior to T cell re‐infusion. For certain blood cancers, namely pediatric B‐cell lymphoblastic leukemia (B‐ALL) and adult aggressive B‐cell lymphomas, ACT has recently entered the standard of medical care.^29^, ^30^, ^31^ In these indications, T cells are genetically modified with a synthetic antigen receptor derived from a monoclonal antibody termed a chimeric antigen receptor (CAR). By contrast, results applying CAR‐modified T cells to solid malignancies have been comparatively modest. Alternative strategies to redirect T‐cell specificity and cytolytic function are therefore necessary if ACT is to serve a greater role in human cancer treatments.

The T cell receptor (TCR) is the antigen recognition structure physiologically expressed by all T cells. The TCR has complementary, and in some cases superior, properties to CARs which likely will prove essential for therapeutically accessing solid tumors. Unlike CARs, TCRs confer recognition to epitopes derived from proteins residing within any subcellular compartment. This enables TCRs to detect a broad universe of targets, such as neoantigens, cancer germline antigens, and viral oncoproteins. Moreover, because TCRs have evolved to efficiently detect and amplify antigenic signals, these receptors respond to epitope densities many fold smaller than required for CAR‐signaling. In this review, we provide a comprehensive overview of the pre‐clinical and emerging clinical data supporting the use of TCR‐based immunotherapies for the treatment of patients with advanced solid cancers. We highlight multiple examples of where TCR‐based immunotherapies have induced durable responses in patients with otherwise treatment refractory cancers. This includes cancers which were previously refractory to non‐specific immunotherapies, such as immune checkpoint inhibitors. These trials have simultaneously highlighted the potential for resistance to TCR‐based therapies to occur. We discuss viable strategies of how to overcome resistance based on resolving the specific mechanism operating in a patient. Finally, we conclude by discussing how TCR‐based immunotherapies will achieve broader dissemination through innovations in clinical next‐generation sequencing, cell manufacturing, and non‐viral genome integration techniques.

## 2 CARS AND TCRS ARE STRUCTURALLY AND BIOPHYSICALLY DISTINCT ANTIGEN RECEPTORS

Clinically, both CARs and TCRs can re‐direct the antigen specificity of lymphocytes when genetically introduced either through transient RNA electroporation^32^ or stable genome integration using viral^33^, ^34^, ^35^, ^36^ and non‐viral approaches.^37^, ^38^ Despite certain similarities, these two classes of antigen receptors are distinct with respect to structure, affinity, immune synapse organization, and the site density of target antigen required to trigger T cell functions. These differences, in turn, have important implications for how these receptors can best be deployed as effective cancer therapies.

### 2.1 CAR structure

As the name “chimera” implies, a CAR is a wholly synthetic single‐chain antigen receptor composed of four distinct modules: (i) an antigen recognition domain, (ii) an extracellular hinge region, (iii) a transmembrane (TM) domain, and (iv) an intracellular T‐cell signaling domain. This modular design has proven to be remarkably versatile.^39^ A CAR's antigen recognition domain frequently is derived from the variable regions of a monoclonal antibody (MoAb) linked together as a single‐chain variable fragment (scFv). Consistent with the site of action of MoAbs,^40^ a scFv can confer recognition of both cell‐surface and soluble antigens^41^ but not intracellular antigens. In addition to scFvs, non‐antibody‐based approaches have also been used to direct CAR specificity, including natural ligand/receptor pairs. For example, cytokines,^42^ innate immune receptors,^43^ TNF receptor superfamily members,^44^ growth factors,^45^ and structural proteins^46^ have also been successfully employed as CAR antigen recognition domains.

The hinge region of a CAR provides the antigen recognition domain with flexibility and distance from the T cell membrane, facilitating synapse formation with target cells. Hinge sequences have been derived from membrane proximal regions of multiple immune molecules, including CD28,^47^, ^48^ CD8α,^49^ and the constant regions of immunoglobulins.^50^, ^51^, ^52^ In addition to determining whether the antigen recognition domain can physically access its target ligand on an opposing cell, the hinge region may also influence T cell persistence^51^, ^53^ and the likelihood of causing cytokine release syndrome.^54^ The TM domain anchors the CAR to the plasma membrane, bridging the extracellular hinge and antigen recognition domains with the intracellular signaling region. The TM region promotes the stable surface expression of a CAR^55^ and can also influence whether the receptor aggregates with itself or endogenous CD3ζ molecules.^56^, ^57^

Finally, the extracellular and TM regions are covalently linked to an intracellular signaling region capable of triggering T cell activation. The earliest versions of CARs, termed first‐generation CARs,^58^ express only a single CD3ζ endodomain.^59^ The CD3ζ endodomain consists of three immunoreceptor tyrosine‐based activating motifs (ITAMs), a conserved amino acid sequence that creates binding sites for the signaling kinase Zap70 when phosphorylated.^60^ Although receptor ligation of a first‐generation CAR triggers antigen‐specific cytokine release and cytolytic function in vitro, T cell proliferation and survival is unable to be sustained. Subsequent iterations, termed second‐generation CARs, incorporate costimulatory signaling domains proximal to the CD3ζ sequence.^61^ This configuration promotes T cell persistence following serial re‐stimulation in vitro^48^ and augments in vivo anti‐tumor efficacy.^62^ The first costimulatory domain tested preclinically was derived from CD28^52^, ^61^; however, signaling domains from a wide variety of other costimulatory molecules, including CD27, CD134 (OX40), CD137 (4‐1BB), CD154 (CD40L), CD278 (ICOS), and CD244 (2B4) have also been successfully tested.^63^ Third‐generation CARs containing two or more co‐stimulatory domains function but do not systematically appear superior to second‐generation CARs. To date, two CARs have been approved for human use and both are second‐generation CARs targeting the B‐cell lineage differentiation antigen CD19. One contains a CD28ζ configuration (axicabtagene ciloleucel)^30^ while the other contains 41BBζ (tisagenlecleucel).^29^, ^31^ Regulatory approval for second‐generation CARs targeting additional B‐cell lineage markers, most notably BCMA,^64^, ^65^ are anticipated in the coming years.

### 2.2 TCR structure

TCRs are the somatically rearranged, naturally occurring antigen receptors expressed on the surface of all T cells. In contrast with the single‐chain configuration of a CAR, the TCR is a heterodimer comprised most commonly of an α and β chain.^66^ Similar to a CAR, both TCR chains possess a variable antigen binding region, an invariant extracellular constant region, and a TM domain. Unlike a CAR, however, neither TCR chain possesses an intrinsic capacity to signal. Rather, the receptor is dependent on a non‐covalent complex of accessory signaling molecules to induce T cell activation. The two TCR chains become covalently linked through a single disulfide bond facilitated by conserved cysteine residues located in each chain's constant region.

Analogous to the variable regions of a MoAb, each TCR chain's variable domain consists of the following regions: (i) a germline‐encoded sequence recombined from a highly polymorphic family of variable (V) and diversity (D) alleles, and (ii) a series of three non‐consecutive hypervariable loops termed complementarity‐determining regions (CDRs). In addition to the V/D alleles, the β chain also possesses a junctional (J) allele to generate even greater diversity. The CDR loops project from each TCR chain and physically contact portions of the MHC molecule alone or in complex with a peptide. The centrally located CDR3 loops are most hypervariable by virtue of somatic rearrangement, dominate the interactions with the peptide, and therefore often contribute to the fine specificity of a TCR for a specific peptide.^66^, ^67^ By contrast, the outward‐facing and germline‐encoded CDR1 and CDR2 loops provide a basal level of TCR affinity for generic MHC molecules through relatively conserved interactions, although CDR1 can contact and contribute to peptide specificity. To initiate downstream signaling, the TCR forms a non‐covalent oligomeric complex with three dimeric CD3 signaling molecules: a CD3ζ homodimer and heterodimers of CD3δε and CD3γε.^68^ In contrast with the signaling domains of most second‐generation CARs, the full TCR complex contains a total of 10 ITAMs.

### 2.3 Antigen recognition by CARs and TCRs

**Figure 1:**
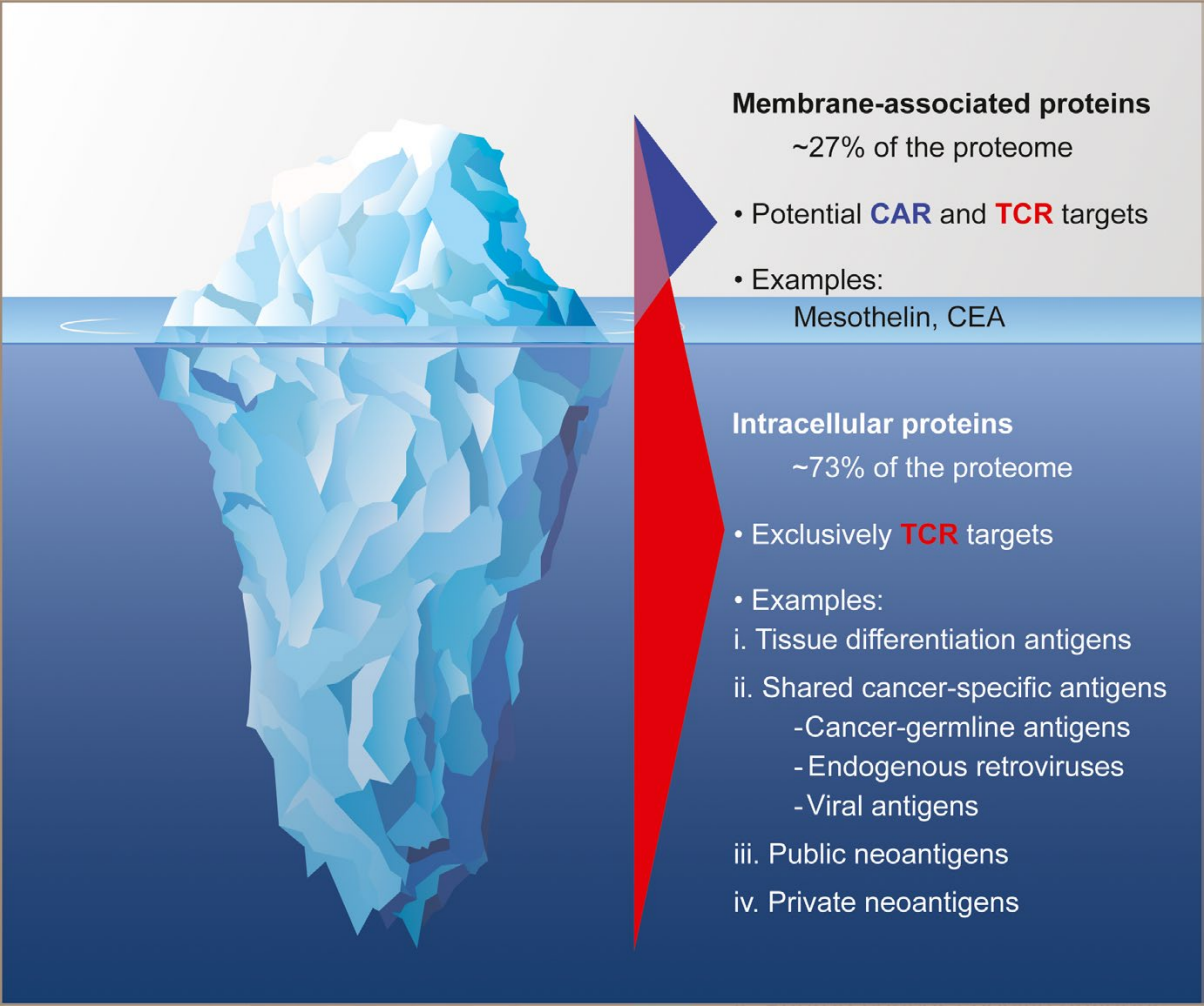
T cell receptors (TCRs) recognize a larger universe of protein‐derived antigens compared with chimeric antigen receptors (CARs). Membrane‐associated proteins, which collectively represent ~27% of the human proteome, can be targeted by both CAR and TCR‐based immunotherapies. TCRs, by contrast, can also target intracellular targets, including cytoplasmic and intra‐nuclear proteins. TCR epitopes are derived from a diverse variety of antigen classes, including tissue‐differentiation antigens, cancer germline antigens, viral oncoproteins, and mutated antigens (cancer neoantigens)

The mechanism by which a CAR molecule binds its target antigen and subsequently triggers T cell activation is distinct from that of a TCR. As noted above, the targets of MoAb‐derived therapeutics, including scFvs, are primarily restricted to cell surface structures.^40^ CARs can directly recognize diverse antigens in an MHC‐independent manner, including unmodified proteins, glycoproteins,^69^ glycolipids,^70^ and carbohydrates.^71^ Current bioinformatic predictions suggest that ~27% of the human proteome contains membrane‐integral structures that might be represented on the cell surface^72^, ^73^ and therefore amenable to CAR recognition (Figure 1). This means that a significant number of potential targets expressed within cancer cells are inaccessible to most CAR‐based therapies. By contrast, TCRs have evolved to recognize epitopes derived from the entirety of the proteome, including cell surface, cytosolic, and intra‐nuclear proteins. Consequently, TCRs may recognize a larger universe of protein‐based targets relative to CARs.

**Figure 2:**
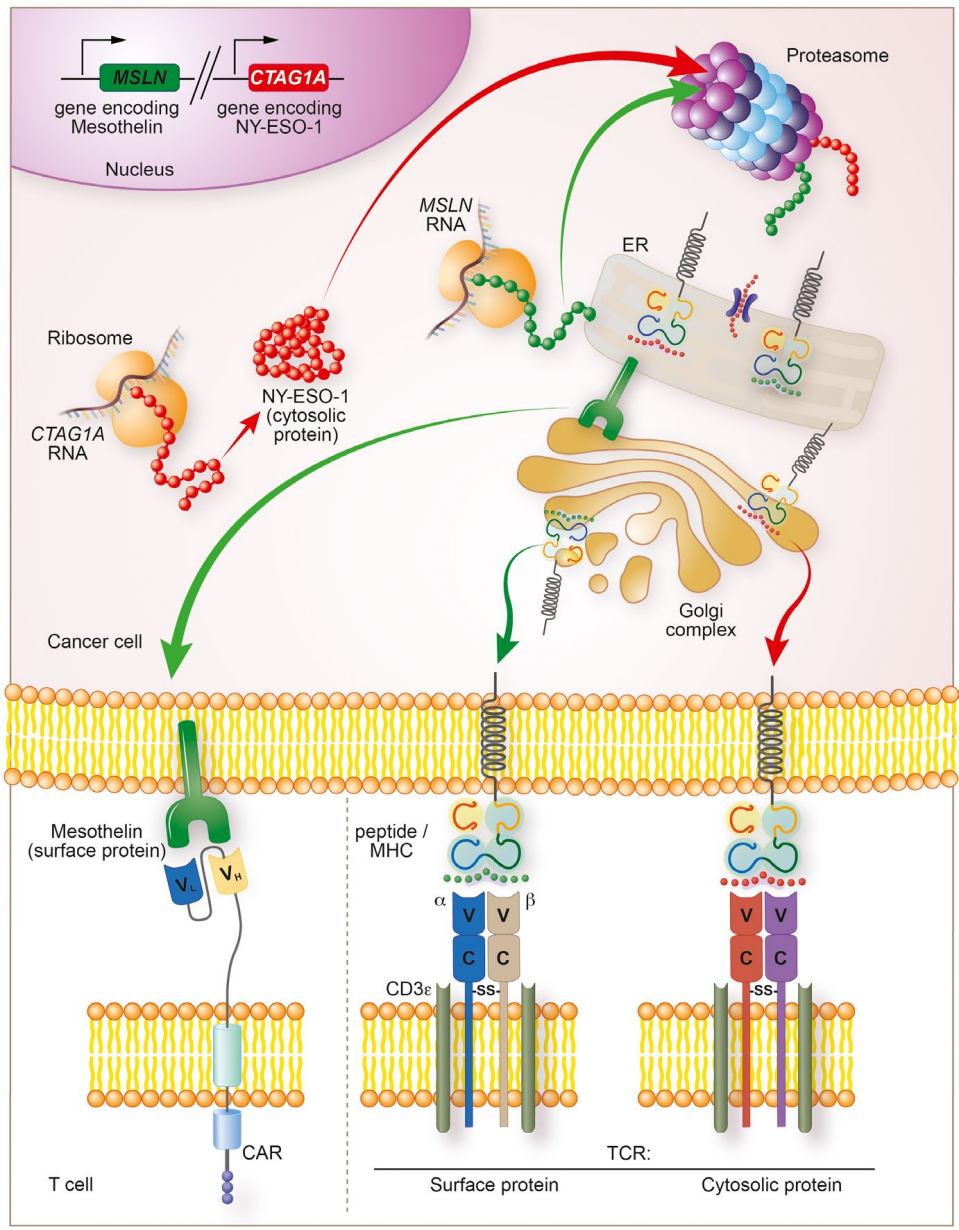
Processing, presentation, and detection of cancer‐associated antigens by chimeric antigen receptors (CARs) and T cell receptors (TCRs). Antigen processing and presentation is a continuous process in most nucleated cells, including cancer cells. This process starts with the transcription of genes encoding proteins that may be destined either for the cell surface membrane (eg, mesothelin) or the cytosol (eg, NY‐ESO‐1). Ribosomes translate transcribed RNA into proteins in the cytoplasm which are then shuttled directly into the endoplasmic reticulum (ER) for proper folding or into the proteasome for degradation. The proteasome generates linear peptide fragments that are transported into the ER via a transporter associated with antigen presentation and loaded on to a major histocompatibility complex (MHC) molecule. Newly folded surface proteins and loaded peptide/MHC (pMHC) complexes then move into the Golgi complex where they are exported to the cell surface. CARs exclusively recognize cell surface proteins; distinct TCRs can recognize pMHC complexes derived from either the cell surface or intracellular proteins. *MSLN* = gene encoding Mesothelin, *CTAG1A* = gene encoding NY‐ESO‐1; V_L_ = variable light chain; V_H_ = variable heavy chain; ‐SS‐ = disulfide bond

Unlike scFvs, the TCR does not bind its cognate antigen directly. In the case of TCRs expressed by CD8^+^ T cells, the ligand is most commonly an 8‐11 amino acid linear peptide sequence presented in complex with one of the patient's complement of major histocompatibility complex (MHC) class I molecules.^74^ Generation of TCR ligands is an ongoing and well‐coordinated process that occurs in most nucleated cells, including cancer cells (Figure 2).^75^ Cellular proteins, whether intracellular or membrane‐associated, are continuously shuttled into the proteasome for degradation into small peptide fragments before release into the cytosol. The resulting peptide fragments are then transported into the endoplasmic reticulum (ER) via a transporter associated with antigen presentation (TAP). Here, peptides with favorable biophysical binding attributes to one of the patient's polymorphic MHC class I molecules form a stable complex (pMHC). The pMHC is then transported to the cell surface via the Golgi apparatus for continuous surveillance by T cells. The net result of these antigen processing and presentation steps is that a TCR is capable of perceiving intracellular antigens displayed on the exterior of a cell. As discussed in detail below, loss of function mutations or epigenetic silencing of any gene involved in these steps may fatally impair the ability of a TCR to function, providing a pathway to therapeutic resistance.

### 2.4 Receptor affinity of CARs and TCRs

CARs that use scFv recognition domains typically have measured affinities in the nanomolar range.^65^, ^76^, ^77^ Native TCRs, by comparison, have affinities several orders of magnitude smaller, typically in the micromolar range,^78^ although nanomolar and picomolar variants have been artificially generated.^79^ In addition to the TCR itself, T cells also express either the CD4 or CD8 co‐receptors. The co‐receptors bind conserved motifs in the MHC molecule where they serve to stabilize TCR/pMHC interactions^80^, ^81^ without directly binding to the presented peptide itself. Additionally, they also recruit proximal signaling kinases that phosphorylate CD3 ITAMs, such as Lck.^82^, ^83^ It is presently unknown whether the co‐receptors influence CAR‐signaling.

### 2.5 CARs and TCRs form distinct immunologic synapses

The immunologic synapse (IS) is the site of interface between an antigen receptor‐bearing T cell and a target cell expressing cognate antigen. The IS serves two essential functions: (i) organizing antigen receptor signaling machinery to amplify and coordinate changes in T cell function and gene expression, and (ii) allowing for directed secretion of noxious cytokines and cytolytic molecules towards target cells.^84^ The IS formed with a CAR is both structurally and temporally distinct from that of a TCR. In conventional TCR/pMHC interactions, the IS morphologically resembles a “bull's eye” pattern consisting of three distinct concentric regions termed supramolecular activation clusters (SMACs).^85^ Each SMAC region contributes to a specific function within the IS. The outermost ring, termed the distal SMAC (dSMAC), is the site where TCR activation initially occurs and is enriched in F‐actin and the protein tyrosine phosphatase CD45.^86^ Following activation, individual TCR microclusters move centrally away from CD45, permitting stable CD3 ITAM phosphorylation and initiation of downstream TCR signaling.^87^ The peripheral SMAC (pSMAC) is contained within the boundaries of the dSMAC and is enriched in the integrin LFA‐1.^85^ The pSMAC stabilizes the IS through interactions between LFA‐1 on T cells and ICAM‐1 expressed on target cells. Finally, the central SMAC (cSMAC) is the site where cytotoxic granules are delivered in a polarized fashion toward the target cell and TCR‐signaling is terminated.^88^ Compared with TCRs, ligation of a CAR results in disorganized antigen receptor and signaling molecule clusters characterized by the punctate accumulation of Lck.^89^ Downstream signal transduction with a CAR is rapid, transient, and less dependent on LFA‐1 relative to a TCR. However, CAR‐mediated killing appears to become attenuated more rapidly than with a TCR, a finding associated with faster downregulation of the CAR.^90^ Although comparatively less well studied than with TCRs, the quality of the IS formed with a CAR has recently been correlated with in vivo anti‐tumor efficacy.^91^, ^92^

### 2.6 CARs require greater antigen site density than TCRs

While scFv‐based CARs have an intrinsically higher affinity for their target antigen relative to a TCR, a TCR can perceive and respond to far fewer molecules on a target cell. To permit comparisons in the signaling efficiency of different receptor classes, it is essential to measure the same experimental endpoint (eg, cytokine release versus cytolysis). TCRs maintain a hierarchical threshold of antigen density to induce different functions, such that greater antigen levels are required to trigger cytokine release compared with cytolysis.^93^, ^94^ TCRs have been shown to induce antigen‐specific cytokine release in response to as little as one pMHC complex.^95^ By contrast, cytokine release from CAR‐modified T cells requires an antigen site density several orders of magnitude greater. For example, an ALK‐specific 41BBζ CAR,^96^ an anti‐CD20 CD28ζ CAR,^97^ and an anti‐CD22 41BBζ CAR^98^ each require between 1,875 and >5,000 target molecules/cell to release cytokine. The mechanism for the discrepancy in the efficiency of TCRs and CARs to trigger downstream function is incompletely understood. One explanation may relate to the ability of relatively lower affinity TCRs to serially engage a single pMHC due to their faster off‐rate versus a scFv‐based CAR. Serial engagement by the TCR may serve to amplify signal.^99^

Independent of antigen receptor affinity, it also appears that the TCR complex itself signals more efficiently than a CAR. Similar to the scFv of a CAR, single‐chain TCRs (scTCRs) can be constructed by covalently linking the variable regions of the TCRα and β chains using a flexible peptide linker.^100^ The scTCR, in turn, can be joined to a truncated TCR constant domain to promote surface expression and membrane tethering.^101^ Like a CAR, a three domain scTCR requires fusion to signaling domains, such as CD3ζ alone or in combination with a costimulatory domain, to trigger T cell effector functions.^102^, ^103^, ^104^ Also analogous to CARs, three domain scTCRs are less efficient compared with conventional TCR heterodimers, requiring higher antigen densities to activate.^103^, ^104^ However, co‐expression of both TCR constant regions together with a scTCR can remove the requirement for a linked signaling domain and restore the functional avidity of the scTCR to approximate the native TCR.^105^ These findings suggest that the natural, non‐covalently linked signaling complex of a TCR is intrinsically more efficient than the linked signaling configuration associated with CARs.

## 3 ENGINEERING EXOGENOUS TCRS TO ENHANCE SAFETY, FUNCTION, AND FEASIBILITY

Detailed understanding of the molecular architecture of the native TCR has permitted rational modifications to its structure, enhancing the safety, efficacy, and scalability of TCR‐based immunotherapies. In the following sections, we discuss how challenges of stably inserting exogenous TCRs into T cells may be overcome through innovations in genetic engineering and structural biology.

### 3.1 TCR mispairing reduces function and potentially compromises safety

**Figure 3:**
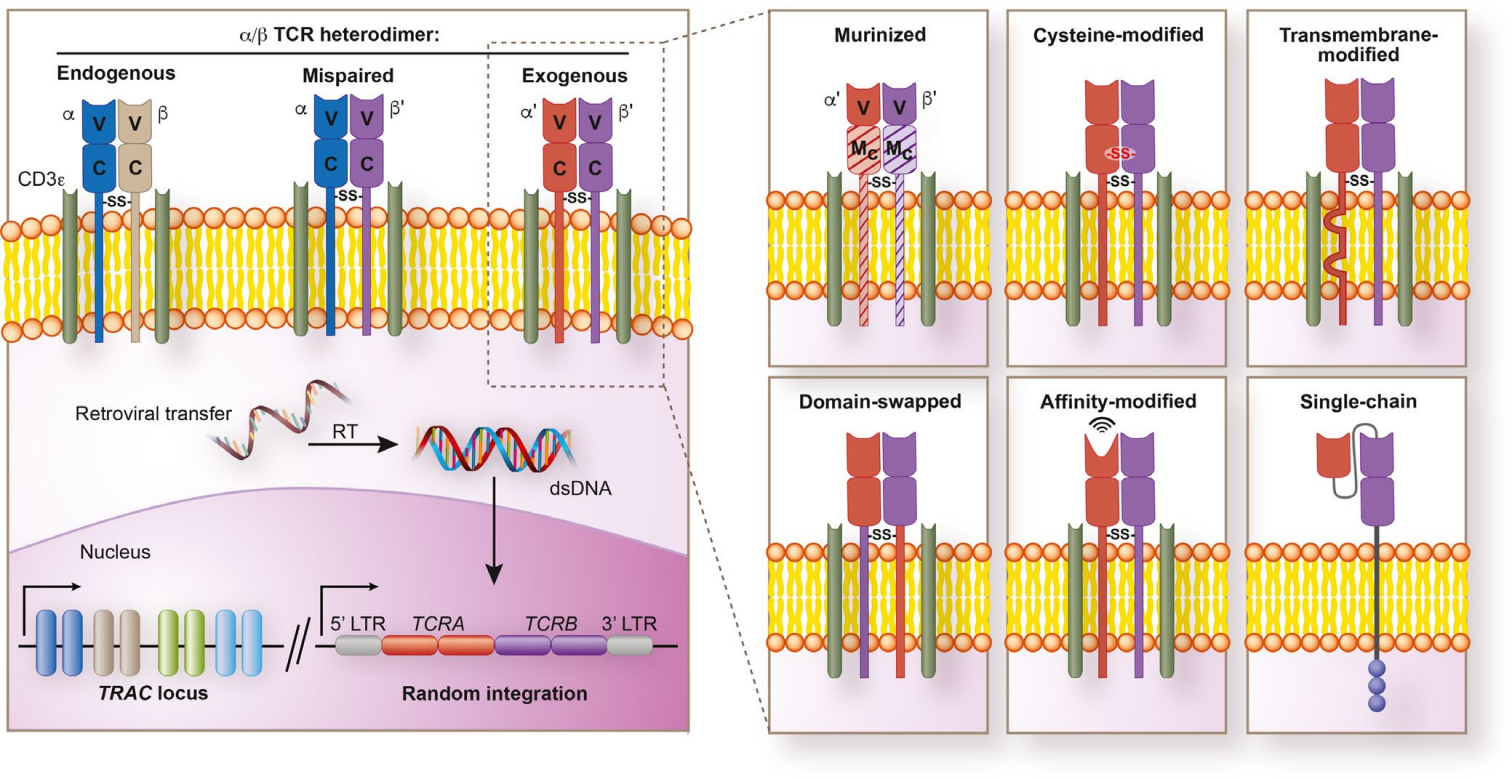
Viral transfer of an exogenous cancer‐specific T cell receptor (TCR) and TCR modifications to enhance both safety and functionality. Double stranded (ds) DNA encoding an exogenous TCR are randomly integrated into the genome of a donor T cell by viral vectors, including γ‐retroviral (shown) and lentiviral vectors. The endogenous α/β TCR, encoded by the *TCR* locus, remains intact and continues to be expressed. As a result, multiple TCR heterodimers may be expressed on an individual T cell's surface, including a mixture of properly paired endogenous (α/β) and exogenous (α’/β’) TCRs as well as mispaired (α/β’ or α’/β) TCRs incorporating chains from both receptors. Mispaired TCRs can reduce the function of properly paired TCRs through competition for signaling molecules, such as CD3ζ, and may also generate new specificities capable of inducing graft versus host disease. The structure of the exogenous TCR may be modified in a variety of ways to either enhance safety and/or augment function. These include: substitution of all or selected murine residues in place of the human sequence in the TCR constant regions (murinization); addition of an extra cysteine residue to promote a second disulfide bond (cysteine‐modification); modification of the hydrophobicity of the TCRα TM region (transmembrane‐modification); inversion of the human sequences of the TCR α and β constant domains (domain‐swapping); mutagenesis of the complementarity‐determining regions loops (affinity‐enhancement); consolidation of a normal TCR heterodimer into a single‐chain format by covalently linking the variable domains of the TCR chains (single‐chain). V = variable region; C = constant region; M_C_ = murinized constant region; RT = reverse transcriptase; ‐SS‐ = disulfide bond

Following viral transfer of TCR genes, the composition of TCRs expressed on a T cell's surface can be a mixture of properly paired endogenous (α/β) and exogenous (α'/β') TCR heterodimers as well as mispaired heterodimers (α/β' and α'/β) (Figure 3). These mixed heterodimers are generated outside thymic selection and therefore remain un‐vetted for the possibility of producing reactivity to self‐antigens. Mouse experiments have demonstrated that formation of mixed TCR heterodimers can cause lethal graft versus host disease (GVHD) following adoptive transfer into syngeneic hosts.^106^ Similarly, generation of mixed TCR heterodimers with allogeneic‐HLA reactivity have also been observed in vitro using human T cells genetically engineered with exogenous TCRs.^107^ Fortunately, no incidence of unexpected GVHD‐like toxicity have been documented in humans receiving TCR‐engineered cell products to date.^108^ In addition to potential safety concerns, TCR mispairing may also compromise the function of an exogenous TCR through competition for signal transduction molecules such as CD3.^109^, ^110^, ^111^, ^112^

### 3.2 Minimizing TCR mispairing through structural modifications

To minimize mispairing between the exogenous and endogenous TCR chains, hybrid TCRs can be created through genetic modifications to the extracellular constant chains. TCR murinization is the substitution of all^109^ or selected^113^, ^114^ murine residues in place of the human sequence in the TCR constant regions. Murinized TCR chains preferentially pair and form more stable complexes with CD3ζ. Together, these properties result in higher exogenous TCR surface expression, increased functional avidity, and enhanced antigen‐specific effector functions compared with a fully human receptor. Similarly, inversion of some^115^ or all^116^ of the human sequences of the TCR α and β constant domains (domain swapping) can also minimize mispairing.

Murine gene sequences can potentially elicit a host immune response following transfer into human patients. Indeed, development of T cell responses to epitopes within the murine‐derived scFv of an anti‐CD19 CAR has occured.^117^, ^118^ Patients who develop an anti‐scFv T cell response lack persistence of CAR‐modified cells and demonstrate worse clinical outcomes. Inclusion of fludarabine in the preconditioning regimen appears to limit the development of an anti‐transgene T cell response, likely by suppressing the host's ability to mount an adaptive immune response. Formation of a humoral immune response to a murine TCR has been observed in clinical trials,^119^ which in some cases appears to sterically block antigen‐specific T cell function. However, development of an anti‐transgene antibody response did not correlate with either T cell persistence or response to treatment. Moreover, epitope mapping revealed the anti‐murine TCR antibodies were directed against the variable region and not the constant chain of the receptor. Taken together, these findings suggest that murinization of the TCR constant region can enhance both the safety and function of exogenous TCRs without compromising T cell persistence.

A second extracellular strategy to reduce TCR mispairing is the introduction of two complementary cysteine residues in the α and β constant regions to promote formation of a second interchain disulfide bond.^120^, ^121^ Similar to murinization and domain swapping, this modification results in enhanced pairing of exogenous TCR chains. Finally, modifying the hydrophobicity of the TCRα TM region by substitution of three aliphatic residues in place of naturally occurring positively charged residues can also stabilize TCR surface expression.^122^ Each of the TCR pairing strategies listed above need not be used in isolation. Indeed, a TCR construct which incorporates all three classes of modifications (murinization, additional cysteine residue, TM hydrophobicity) has recently entered a first in human clinical trial (NCT02858310).^123^ While in principle concerns regarding TCR chain mispairing may be mitigated by condensing the structure of the TCR heterodimer into a scTCR format, no scTCR constructs have been clinically tested.

### 3.3 TCR‐affinity enhancement

Several groups have tested the impact of inserting mutations in the CDR loops on human TCR affinity and function. Amino acid substitutions can be made either empirically^124^, ^125^, ^126^ or through directed evolution using phage‐display libraries.^79^ Substitutions within the CDR3 loops have been the focus of most investigations^79^, ^124^, ^125^, ^126^, ^127^; however, mutations in the CDR1 and CDR2 loops can also lead to augmented TCR‐MHC interactions.^79^, ^128^, ^129^ Because affinity‐enhanced TCRs have not undergone normal thymic development, unintended cross‐reactivity to self‐antigens may be generated. As will be discussed in detail below, ACT using T cells genetically engineered with affinity‐enhanced TCRs have resulted in lethal off‐tumor/off‐target toxicities in two clinical trials.^130^, ^131^ Detailed analyses from both trials revealed that the affinity‐enhanced TCRs exhibited unanticipated cross‐reactivity to antigens expressed in critical normal tissues.^127^, ^130^, ^131^ Consequently, testing for potential cross‐reactivity is now routinely performed during the pre‐clinical development of novel TCRs.^123^, ^132^, ^133^, ^134^ It is important to note, however, that development of off‐tumor/off‐target toxicities is by no means a universal property of all affinity‐enhanced TCRs. Indeed, an affinity‐enhanced anti‐NY‐ESO‐1 TCR has demonstrated exceptional anti‐tumor efficacy in multiple clinical trials without evidence of off‐target toxicities.^135^, ^136^, ^137^, ^138^

### 3.4 Non‐viral TCR integration strategies

Retro‐ and lenti‐viral vectors have been the primary means of stably inserting transgenes encoding exogenous TCRs into T cells for clinical application. Although viral‐based approaches have been effective in generating therapeutic T cell products, they are not without several important limitations. First, integrating viral vectors can be associated with additional safety concerns beyond those associated with the formation of mixed TCR heterodimers. Viral vectors integrate semi‐randomly into a T cell's genome, often with a bias toward transcriptionally active genes.^139^ To date, no transformation events have been recorded following viral transfer into mature T cells.^140^ Nevertheless, it was recently reported that a patient receiving lentiviral‐modified T cells developed clonal repopulation with central memory T cells (T_CM_),^141^ a minimally differentiated T cell subset with enhanced survival capacity.^142^, ^143^ Integration site analysis revealed that the virus disrupted the function of *TET2*, a gene involved in epigenetic modification which is also associated with myeloid malignancies.^144^ This finding highlights that viral integration‐induced gene disruption remains a clinical possibility, albeit a rare one. Second, integrating viruses leave the endogenous TCR intact, providing a “sink” for limiting intracellular signal transduction molecules, including CD3 and Lck. Finally, virally integrated sequences can be associated with variegated gene expression, leading some T cells to express high levels of an antigen receptor while others express minimal or none.^145^ This can produce variability, both in the potency and toxicity profiles of individual cell products using the same antigen receptor.

**Figure 4:**
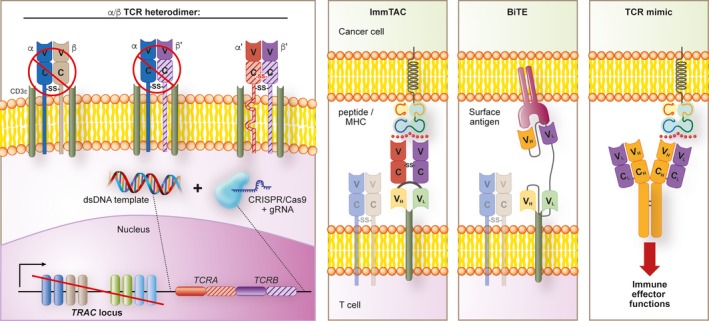
Targeted T cell receptor (TCR) delivery and TCR‐like structures. CRISPR/Cas9‐mediated TCR delivery can direct the targeted genomic replacement of an exogenous TCR into the endogenous *TRAC* locus. Disruption of the endogenous TCR eliminates expression of endogenous and mispaired TCRs. The exogenous TCR is homogenously and stably expressed under the endogenous *TRAC* promoter on the cell surface. TCR‐like structures, including bispecific soluble TCRs (immune‐mobilizing monoclonal TCRs against cancer; ImmTAC), bispecific antibody‐like structures (Bispecific T‐cell Engagers; BiTE), and antibodies specific for peptide/MHC complexes (TCR mimics) provide alternative approaches to re‐direct T cell specificities to tumor antigens without the need for genomic integration. V = variable region; C = constant region; V_L_ = variable light chain; V_H_ = variable heavy chain; C_L_ = constant light chain; C_H_ = constant heavy chain ‐SS‐ = disulfide bond

Targeted integration into genomic “safe harbors”,^139^ including the endogenous *TCR* locus, can overcome many of these challenges (Figure 4). First, integration at a defined site removes concerns about the possibility of disrupting normal gene functions that restrain cellular immortalization and transformation. Second, disruption of the endogenous TCR eliminates the possibilities of TCR mispairing and competition for TCR signaling components. Third, TCR integration into the *TCR* locus places expression of the receptor under physiologic transcriptional control, potentially minimizing tonic signaling and immunologic exhaustion.^36^ Finally, targeted integration using a fully non‐viral method can reduce clinical manufacturing costs as GMP nucleic acids are less expensive to produce than viral particles.

Recently, two groups demonstrated the feasibility of knocking in an exogenous TCR into the human *TCR* locus using CRISPR/Cas9.^38^, ^146^ Importantly, in work by Roth et al,^38^ both disruption of the *TCR* locus and replacement with an exogenous TCR template was achieved using an entirely non‐viral approach. This was accomplished through co‐electroporation of a guide‐RNA loaded CRISPR‐Cas9 ribonucleoprotein (RNP) complex in combination with a long double stranded DNA template. In a second example, disruption of the *TCR* locus was also achieved through electroporation with a CRISPR‐Cas9 RNP.^146^ However, the donor DNA template was provided through infection with an adeno‐associated virus (AAV)6 viral transfer system, building upon earlier work by Eyquem et al using CARs.^36^ Both the fully non‐viral and AAV6 methods generated T cells with a reasonable frequency of genome integration, minimal to no genomic off‐target effects, good cell viability, and evidence of in vivo anti‐tumor efficacy.

### 3.5 Soluble TCRs, T‐cell engaging bispecifics, and TCR‐mimetics

Recent innovations in protein engineering are enabling use of TCR and TCR‐like antigen recognition structures for therapeutic purposes without the need for gene engineering and ACT (Figure 4). Because these “off the shelf” reagents do not require manufacturing for individual patients, they offer the potential for wider and more streamlined clinical deployment. For example, immune‐mobilizing monoclonal TCRs against cancer (ImmTACs) are fusion proteins that combine a soluble high‐affinity TCR with a scFv‐based anti‐CD3 binding domain.^147^ ImmTACs can redirect the cytolytic function of non‐specific T cells by engaging a cognate pMHC complex on target cells and triggering activation of adjacent T cells through CD3 cross‐linking. A clinical trial using an ImmTAC specific for the shared self/tumor antigen gp100 has shown promising activity in uveal melanoma, an immune checkpoint inhibitor refractory cancer.^148^

Bispecific T‐cell Engagers (BiTEs) and TCR mimics are two additional classes of recombinant proteins that also confer antigen‐specific immune targeting without the need for ex vivo cell manipulation. Unlike ImmTACs, both BiTEs and TCR mimics exclusively utilize antibody‐based scFv domains for antigen recognition. Similar to ImmTACs, BiTEs are bispecific fusion proteins that contain two unique antigen binding domains.^149^ One scFv is used for binding to antigen‐expressing target cells while the other cross‐links CD3. Based on this mechanism of action, BiTEs can activate T cells in a MHC‐independent manner. Blinitumimab, a CD19‐directed T‐cell bispecific engager, has entered the standard of care for adult and pediatric patients with pre‐B ALL.^150^, ^151^ Although no BiTEs are presently approved for solid malignancies, clinical data are beginning to emerge targeting HER2^152^ and CEA^153^ with next‐generation variations of bispecific technology. TCR mimics are structurally identical to conventional MoAbs but have specificity for only a single antigen, in contrast with BiTEs.^154^ Analogous to the recognition motif of TCRs, TCR mimics are specific for linear peptide sequences derived from intracellular proteins and presented in the context of an MHC molecule. Because they are derived from full‐length antibodies, TCR mimics can elicit a diverse array of immune and non‐immune effector functions that are distinct from a conventional T cell response. These include antibody‐, cell‐, and complement‐mediated cytotoxicity^155^ in addition to induction of apoptosis in an immune effector‐independent manner.^156^

## 4 CLINICAL EXPERIENCE WITH TCR‐BASED CANCER IMMUNOTHERAPIES

### 4.1 Elucidating the landscape of cancer‐associated antigens recognized by TCRs

The greatest experience with TCR‐based immunotherapies in solid malignancies to date has been the adoptive transfer of tumor infiltrating lymphocytes (TIL). TIL are obtained from the surgical resection of a cancer metastasis followed by non‐specific ex vivo lymphocyte expansion to treatment numbers (up to 10^11^ cells). Experimentally, TIL derived from a diverse range of human solid cancers demonstrate autologous tumor cell reactivity that can be blocked by anti‐HLA antibodies, implying these lymphocytes express TCRs specific for cancer‐associated antigens. Cancers from which reactive TIL has been expanded include tumors of the breast,^157^ gastrointestinal tract,^158^ head and neck,^159^ kidney,^160^ lung,^161^ and ovary^162^ in addition to cutaneous^5^, ^163^, ^164^, ^165^, ^166^ and uveal melanoma.^13^, ^158^, ^165^ Adoptive transfer of TIL following a lymphocyte‐depleting chemotherapy regimen has led to objective cancer shrinkage in each of these diseases. This includes patients who were previously refractory to or progressed on an immune checkpoint inhibitor.^13^, ^164^, ^166^, ^167^ These observations establish important proof of principle that TCR‐based therapies can mediate regression of solid cancers in patients. However, whereas ~50% of cutaneous melanoma patients respond to TIL therapy,^163^, ^164^, ^166^ only a minority (<15%) of patients with epithelial malignancies show evidence of cancer regression. Efforts to understand the determinants of successful TIL‐based therapies have focused on resolving which classes of antigens are recognized by infiltrating T cells in responding patients. These studies have revealed that TIL can recognize a wide spectrum of antigens, including tissue differentiation antigens,^6^, ^168^ cancer germline antigens,^12^ viral oncoproteins,^12^, ^15^ and neoantigens.^5^, ^6^, ^8^, ^10^, ^11^, ^12^, ^17^, ^169^, ^170^ Despite evidence of clinical activity, broad dissemination of TIL therapies has been limited by practical challenges associated with procuring and expanding T cells from surgically obtained samples. Consequently, significant efforts have been made to develop less invasive methods for generating antigen‐specific T cells from the peripheral blood, including in vitro sensitization (IVS) and TCR gene engineering.

### 4.2 Tissue differentiation antigens

There exist practical reasons for seeking to immunologically target tissue differentiation antigens. These antigens are frequently shared between patients and expressed at high levels by cancer cells. However, TCR trials targeting this class of antigens have demonstrated significant on‐target/off‐tumor toxicities and only modest anti‐tumor activity. The first clinical trials testing the genetic insertion of an exogenous TCR focused on shared tumor/tissue‐differentiation antigens, including MART‐1,^33^, ^171^, ^172^, ^173^ gp100,^171^ and CEA.^174^ Destruction of normal melanocytes in the eye, skin, and inner ear occurred in patients receiving MART‐1 and gp100‐specific TCRs, resulting in uveitis, vitiligo, and auditory/vestibular dysfunction. Similar, albeit less severe, on‐target toxicities have also been observed in patients receiving MART‐1 and gp100‐specific T cells raised through IVS.^175^, ^176^, ^177^, ^178^ In three patients who received CEA‐specific TCR engineered T cells, each developed an inflammatory colitis requiring systemic immune‐suppression with high‐dose steroids and anti‐cytokine antibodies. These findings directly parallel the on‐target but off‐tumor B‐cell aplasia observed with targeting the hematologic shared tumor/tissue‐differentiation antigen CD19 using CARs.^34^ Taken together, these results emphasize the critical need to develop TCRs specific for antigens that are selectively, if not exclusively, expressed by cancer cells and not essential normal tissues.

### 4.3 Cancer germline antigens and endogenous retroviruses

Similar to tissue differentiation antigens, the cancer germline antigens (CGAs) are a class of immunogenic intracellular proteins whose expression can be shared between patients. Unlike the differentiation antigens, however, normal tissue expression of CGAs are confined primarily, although not always exclusively, to germ cells.^179^ Because germ cells lack expression of HLA, they are protected from T cell‐mediated immune injury. Finally, CGAs can be expressed by a diverse variety of cancers, including common epithelial malignancies.^180^ These attributes collectively make the CGAs an attractive group of therapeutic TCR targets.

The family of CGAs is represented by over 100 proteins, the majority of which have gene loci along the X chromosome^179^ where they are negatively regulated by epigenetic silencing.^181^ Among the CGAs, NY‐ESO‐1 and MAGE‐A3 have been the two most commonly tested in ACT TCR clinical trials to date. NY‐ESO‐1 has been targeted using an unmodified endogenous TCR from a HLA‐DPB1*04:01 (DPB1)‐restricted CD4^+^ T cell clone raised by IVS^182^ and gene engineering with an affinity‐enhanced HLA‐A2*01:01 (A2)‐restricted TCR.^135^, ^136^, ^137^, ^138^ Despite mediating cancer regression, including durable CRs, no evidence of off‐tumor toxicity was observed in these trials. Cancer regression has also been observed following ACT of TCR‐engineered lymphocytes targeting members of the MAGE‐A3 family. In one trial, patients received transfer of T cells engineered with a CDR3‐unmodified TCR specific for the MAGE‐A3/6 members and restricted by the MHC class II allele DPB1.^133^ Responses were seen in patients with diverse solid tumors, including cancers of the cervix, bladder, esophagus, and bone.^183^ As with the NY‐ESO‐1 trials, no off‐target toxicities were observed with this receptor.

Two additional trials tested MAGE‐A3 targeting using affinity‐enhanced TCRs. One trial targeted an epitope derived from MAGE‐A3 that was restricted by HLA‐A2.^126^ Of note, the TCR used in this study pre‐clinically demonstrated cross‐reactivity to a closely related epitope derived from MAGE‐A12. Clinically, responses were seen in 5/9 (56%) of patients, including one patient who achieved a CR after previously progressing following infusion of NY‐ESO‐1 specific T cells.^130^ However, in this same trial, lethal neurologic toxicities occurred in two patients and a third experienced seizures and mental status changes that resolved with immune suppression. A detailed post‐event analysis revealed neuronal expression of MAGE‐A12, a previously unknown finding that likely explained the toxicities observed in this study. Lethal off‐target/off‐tumor toxicities were also observed in a second trial that tested an affinity‐enhanced TCR reactive against a MAGE‐A3 epitope restricted by HLA‐A1.^127^ In this trial, 2/2 patients developed fulminant cardiac toxicity within days of receiving TCR‐engineered T cells.^131^ Investigations following these adverse events discovered that the TCR cross‐reacted to an epitope derived from Titin, a striated‐muscle protein expressed in the myocardium. These clinical data emphasize the critical importance of assessing TCR cross‐reactivity prior to clinical development, especially when using affinity‐enhanced TCRs that have not undergone thymic selection. However, they also demonstrate the potential utility the large family of CGAs can have in mediating cancer regression in humans.

Like CGAs, the human endogenous retroviruses (H‐ERVs) represent a second class of immunogenic proteins that are shared between patients and epigenetically silenced in normal tissues.^184^ H‐ERVs are the degraded remnants of retroviral gene sequences that integrated into the human germline in the distant past and which today account for ~8% of the genome. The H‐ERVs rarely are expressed in most normal tissues except for periods of epigenetic dysregulation, such as malignant transformation.^185^ The protein products of H‐ERV genes can give rise to epitopes presented in the context of HLA molecules. T cells specific for H‐ERV‐derived epitopes are capable of lysing cancer cells in vitro, including cancers of the breast,^186^ kidney,^187^ ovary,^188^ and melanoma.^189^ Careful studies assessing the uniformity of H‐ERV protein expression in tumor masses and normal tissue have yet to be performed, a prerequisite for clinical development. Nevertheless, because H‐ERVs have the unique attribute of triggering a strong type‐I IFN response through viral mimicry,^190^ further exploration of this class of TCR targets is warranted.

### 4.4 Virus‐derived oncoproteins

Cancers may also express antigens from more recently integrated oncogenic viruses. In the case of solid malignancies, these can include high‐risk strains of the human papillomavirus (HPV), the hepatitis B and C viruses, the Merkel cell polyomavirus (MCPyV), and Epstein Barr virus (EBV). The potential advantages of immunologically targeting epitopes derived from viral oncoproteins are several‐fold. First, these proteins are immunologically foreign and therefore not expressed in the thymus or uninfected normal tissues. Consequently, the T cell repertoire to such antigens should be of relatively high affinity compared with self‐antigens^191^ and the therapeutic window between normal and diseased tissue wide. Second, because the oncoproteins directly contribute to the malignant phenotype, expression of these antigens should be conserved between metastases and development of antigen loss variants minimized. Finally, because ~12% of human cancers result from infection with an oncogenic virus,^192^ targeting this class of antigens can potentially benefit a significant population of patients using a common set of reagents.

In the case of HPV, the E6 and E7 oncoproteins are constitutively expressed and clonally maintained within patients.^193^ Two HLA‐A2‐restricted TCRs specific for epitopes derived from the HPV‐16 E6^132^ and E7^123^ oncoproteins have entered first in human clinical trials (NCT02280811 and NCT02858310). Preliminary results suggest that both TCRs are active and capable of inducing objective responses in patients with HPV‐associated cervical, anal, and head and neck cancers. Analogous to the HPV E6/E7 antigens, Merkel cell carcinoma associated with MCPyV infection constitutively expresses the viral large T‐antigen (LTAg) oncoprotein. Adoptive transfer of autologous MCPyV LTAg‐specific T cells can also induce objective responses in patients with metastatic disease.^194^, ^195^ Finally, ACT of T cells specific for the EBV‐associated oncoprotein LMP1 have also successfully mediated cancer regression in patients with nasopharyngeal carcinoma.^196^ In each of these examples, no off‐target/off‐tumor toxicities were observed. Collectively, these data provide evidence that oncogenic virus‐associated antigens can be a valuable source of cancer regression antigens.

### 4.5 Public and private cancer neoantigens

A final class of antigens, termed cancer neoantigens,^197^ are derived from somatic genomic events resulting in non‐synonymous point mutations,^4^, ^5^, ^6^, ^8^, ^12^, ^18^, ^198^ insertions,^199^ gene fusions,^200^ or alternative RNA editing.^201^ Because neoantigens are exclusive to tumor cells, the risk of on‐target/off‐tumor injury to healthy tissues is minimized. Additionally, because neoantigens are not germline‐encoded and therefore absent from the thymus, the TCR repertoire to such antigens should be broad and of relatively high affinity. The mutational landscape of cancer can vary greatly, both between different cancer types as well as patients.^202^ Nevertheless, certain evolutionary patterns exist. Mutations in driver oncogenes and tumor‐suppressor genes that directly contribute to the malignant phenotype frequently appear early and tend to be clonally shared between metastases.^203^ Function‐altering mutations typically occur in constrained hotspot regions shared between patients, often with one or a limited number of amino acid substitutions. Additional passenger mutations, including those appearing as a consequence of DNA damage from cytotoxic or radiation treatments, are typically (but not always) subclonal and held in private by individual patients.^204^ From the lens of TCR targets, the distinction between shared driver mutations and private passenger mutations has immunologic consequences.

A public neoantigen is a peptide containing a hotspot mutation that is restricted by a relatively common HLA allele.^205^ Although previously thought to be rare,^19^ the number of identified public neoantigens has rapidly increased in recent years. These include public neoantigens associated with common driver oncogenes, such as KRAS,^11^, ^18^, ^206^, ^207^ BRAF,^170^ beta‐catenin,^198^ CDK4,^208^, ^209^ H3.3K27M,^210^ and IDH1^211^ as well as tumor suppressor genes, like TP53.^212^, ^213^ Whether immunogenic epitopes can be discovered for the 119 other known driver oncogenes^214^ remains an area of active investigation. Similar to viral oncoproteins, immunologically targeting public neoantigens offers several advantages. Public neoantigens are shared among patients, enabling the use of common “off the shelf” TCR reagents. Because targeted next‐generation sequencing to identify driver mutations has entered routine clinical use, identification of patients harboring public neoantigens has been streamlined. Finally, because driver oncogenes contribute to oncogenesis, antigen expression should be clonally conserved, minimizing the risk of antigen‐loss variants. Several recent clinical trials have provided in vivo evidence that infusion of public neoantigen‐specific T cells can be associated with durable cancer regression in humans.^11^, ^170^

Notwithstanding these exciting findings, it remains the case that most cancer neoantigens are privately held by individual patients. For example, in a series of 31 patients with metastatic cutaneous melanoma, neoantigen‐specific T cells could be identified in 29/31 (93.5%) of patients.^19^ However, not one of these neoantigens was held in common. Similarly, in a cohort of 35 consecutively evaluated patients with metastatic microsatellite‐stable GI malignancies, neoantigen‐specific reactivity was identified in 31/35 (89%) of patients. Among these, only two patients shared reactivity to a KRAS^G12D^ public neoantigen. Taken together, among 148 empirically identified neoantigens, only 2/148 (1.3%) were shared and 146/148 (98.7%) were patient‐specific. These data highlight two critical conclusions regarding neoantigen targeting: 1) most patients, even those with modestly mutated common epithelial cancers, express immunogenic neoantigens; however, 2) most neoantigens will need to be resolved at the level of individual patients. Elucidating patient‐specific private neoantigens in a manner that is at once rapid, scalable, and cost‐effective represents an unprecedented challenge.^215^ Pre‐clinical data suggests that preparative sorting of TIL or PBMC based on expression of co‐inhibitory markers (PD1, TIM3, CD39)^159^, ^207^, ^216^, ^217^ or acute activation markers (CD134, CD137)^162^, ^207^, ^216^ can enrich for neoantigen‐specific T cells. ACT of TIL enriched for private neoantigen reactivity has resulted in durable CRs in a subset of patients with common solid cancers, including those arising from the bile duct, breast, and cervix.^8^, ^12^, ^17^

## 5 THERAPEUTIC RESISTANCE TO TCR‐BASED CANCER IMMUNOTHERAPIES

**Figure 5:**
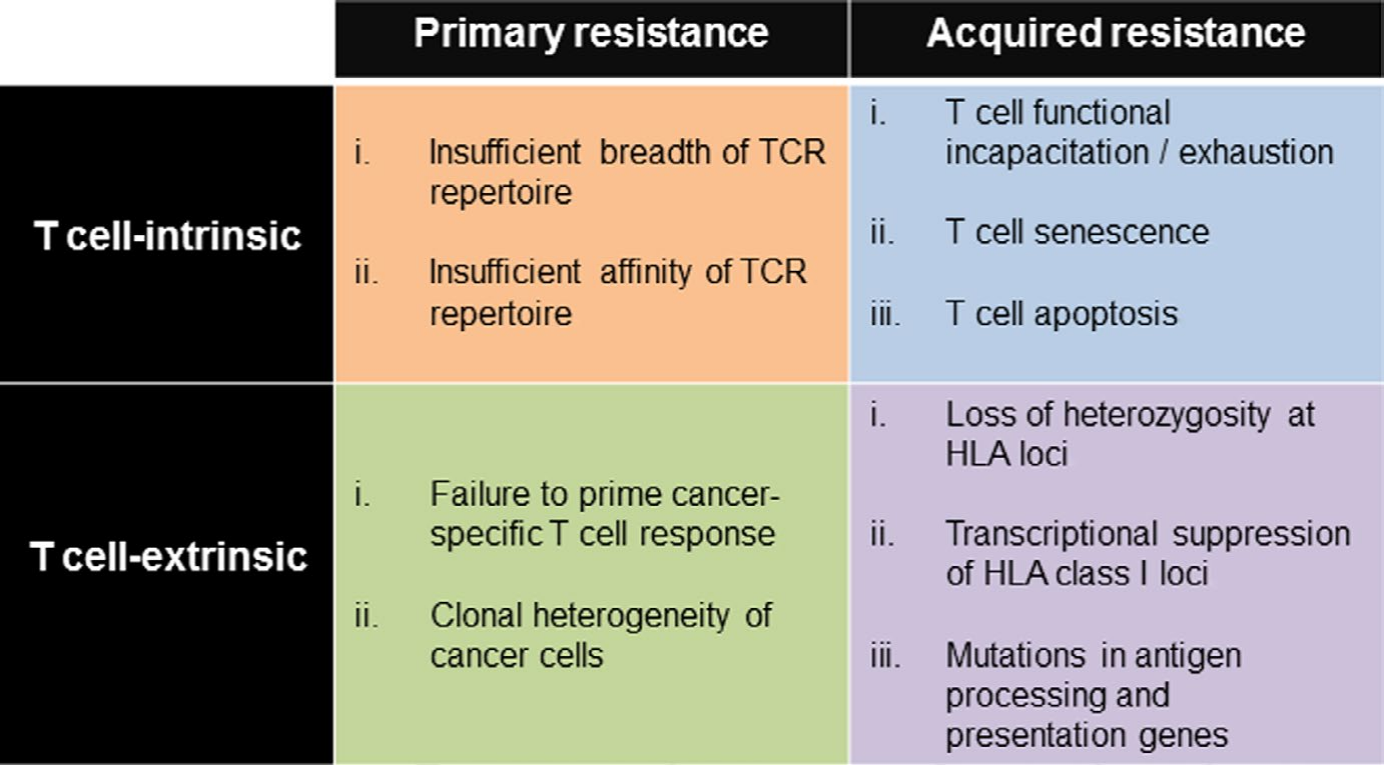
Mechanisms of therapeutic resistance to T cell receptor (TCR)‐based cancer immunotherapies. The mechanistic basis for TCR resistance can be subdivided into the following four categories: primary (1°) versus late/acquired and T cell‐intrinsic versus extrinsic. Strategies to successfully overcome resistance to TCR‐based immunotherapies are possible and are focused on the specific resistance category into which a patient falls

Resistance to TCR‐based immunotherapies is increasingly being recognized as a clinically significant entity.^11^, ^175^, ^194^, ^195^, ^218^, ^219^ Conceptually, the mechanistic basis for TCR resistance can be subdivided into the following four categories: primary (1°) versus late/acquired and T cell‐intrinsic versus extrinsic (Figure 5). In this schema, the distinction between 1° and late/acquired resistance is based solely on whether the patient achieves a clinical response following a TCR therapy. It is important to note, however, that the molecular mechanisms which contribute to these patterns of resistance can overlap among patients in both response categories. Strategies to successfully overcome resistance to TCR‐based immunotherapies are possible and are focused on the specific resistance category into which a patient falls.

### 5.1 Primary T cell‐intrinsic resistance

Primary T cell‐intrinsic resistance occurs when the TCR repertoire is insufficiently broad to either detect or productively respond to the antigens expressed on a cancer cell's surface. This can occur when there is an absence of any TCR within a patient with specificity for a target antigen, including neoantigens,^220^ or when a specific TCR lacks sufficient affinity to mediate cytotoxicity.^221^ Aging^222^ and the lymphodepleting influence of certain cancer therapies, including chemotherapy^24^, ^25^, ^26^ and some forms of radiotherapy,^223^ can reduce the breadth of a patient's TCR repertoire. These factors, in turn, can limit the likelihood that the patient will have T cell clonotypes capable of recognizing cancer cells. Cancers that harbor a low mutational burden can also be associated with this form of 1° resistance. Leukemias, pediatric malignancies, and many epithelial cancers often possess a modest number of somatic mutations and a corresponding limited neoantigen load,^185^ restricting the number of potential TCR targets.^224^ Overcoming primary T cell‐intrinsic resistance is centered on expanding the TCR repertoire. This can be accomplished by the ex vivo introduction of a TCR conferring anti‐tumor specificity into non‐specific T cells through genetic engineering followed by ACT.^215^ Powerful proof of principle of this approach is evident in HLA‐A*02:01^+^ patients with metastatic synovial cell sarcoma receiving adoptive transfer of NY‐ESO‐1 TCR engineered T cells.^135^, ^137^ Synovial cell sarcoma is genomically simple,^225^ typically not associated with a T cell infiltrate,^226^ and largely unresponsive to immune checkpoint inhibitors.^227^, ^228^ Nevertheless, adoptive transfer of NY‐ESO‐1‐specific TCR engineered T cells results in objective cancer regression in ~50%–61% of chemotherapy‐refractory patients. In addition to ACT, overcoming 1° T cell‐intrinsic resistance might also be accomplished through infusion of a recombinant bispecific TCR^229^ and possibly vaccination with patient‐specific T cell neoepitopes.^230^, ^231^, ^232^

### 5.2 Primary T cell‐extrinsic resistance

Primary T cell‐extrinsic resistance arises when a patient's TCR repertoire is sufficient to recognize cancer cells, but additional factors limit the capacity of T cells to mediate clinically apparent cancer regression. This pattern of resistance can occur either before or after T cell priming has occurred. For example, cancers associated with activating mutations in the WNT/β‐catenin pathway can impair T cell priming by restraining the function of antigen cross‐presenting basic leucine zipper transcriptional factor ATF‐like three lineage dendritic cells (BATF3 DCs).^233^ In mice, tumors harboring constitutively active variants of β‐catenin limit BATF3 DC migration to tumor draining lymph nodes, preventing naïve T cell priming and tumor cell infiltration. In contrast, BATF3 DC migration and T cell priming is not impacted when mice are inoculated with otherwise identical tumor lines expressing wild type β‐catenin. A reduced T cell infiltrate is observed in patients whose tumors possess gain of function mutations in the WNT/β‐catenin pathway, suggesting this mechanism of immune evasion is conserved across species.^234^ Similar defects in T cell priming might also occur in cancers associated with mutations in other driver oncogenes, including PIK3CA, or loss of function in tumor suppressor genes such as PTEN.^235^

Alternatively, T cell priming can successfully occur but heterogenous antigen expression can limit the ability of activated T cells to successfully clear all tumor cells. For neoantigens, intra‐ and inter‐tumoral genetic heterogeneity can lead to this form of resistance,^204^ especially when the targeted antigens are derived from passenger mutations. The presence of a significant proportion of genetic subclones within a tumor mass leads to a loss of T cell‐mediated anti‐tumor efficacy in mice.^236^ Similarly, genetic clonal heterogeneity is associated with a lack of treatment efficacy to immune checkpoint inhibitors in humans.^204^ Heterogeneity in the epigenetic landscape of cancer cells,^237^ although less well characterized than genetic heterogeneity, may also contribute to variable antigen expression and clinical TCR resistance. This possibility is exemplified by the cancer germline antigens (CGAs). The pattern of CGA expression by cancer cells can be remarkably variable, both between patients and within individuals. In some cases, tumor cells exhibit strong, uniform expression while in many cases CGA expression is patchy and restricted to only a minor population of cancer cells,^238^, ^239^ suggesting heterogeneity in epigenetic marks. For patients where only a subset of cancer cells express the targeted CGA, a clear path to therapeutic TCR resistance exists through selection pressure for antigen‐negative clones.

Overcoming primary T cell‐extrinsic TCR resistance depends on whether the defect resides at the level of T cell priming versus heterogeneity in antigen expression. In the case of resistance attributable to a T cell priming defect, restitution of BATF3 DC function can promote T cell‐mediated anti‐tumor immunity. This can be accomplished through adoptive transfer of BATF3 DCs,^233^ pharmacologic inhibition of the Wnt/β‐catenin pathway, or administration of STING^240^ or toll‐like receptor (TLR)‐3^241^, ^242^ agonists to promote local BATF3 DC function. Alternatively, the need for in vivo T cell priming can be supplanted altogether by ACT of tumor‐specific T cells. Overcoming antigen heterogeneity is comparatively more challenging. One strategy could be to selectively target clonally conserved neoepitopes, such as public neoantigens, or to simultaneously target multiple private neoantigens. DNA methyl‐transferase inhibitors and other epigenetic modifiers, including histone deacetylase inhibitors, can increase CGA expression in cancer cells but not fibroblasts in vitro.^181^, ^243^ It is presently unknown whether epigenetic modifiers can enforce uniform CGA expression in cancer cells in vivo. Epitope spreading, the development of T cell reactivity to antigens distinct from those initially targeted, has been observed in a subset of patients receiving TCR‐based ACT.^182^, ^244^ However, the frequency with which epitope spreading occurs and the extent to which this phenomenon may overcome antigen heterogeneity remains unclear.

### 5.3 Acquired/late T cell‐intrinsic resistance

Acquired/late T cell‐intrinsic resistance occurs when the TCR repertoire is sufficient to recognize antigens expressed on a cancer cell's surface but the responding T cell population is either functionally incapacitated or incapable of clonal expansion. T cell functional incapacitation, colloquially referred to as exhaustion,^245^ is mediated by the upregulation of surface co‐inhibitory receptors and/or intracellular negative regulators of TCR signaling. Perhaps the best characterized of the co‐inhibitory surface receptors is PD‐1, an activation‐induced transmembrane protein capable of recruiting inhibitory phosphatases such as SHP‐1 and SHP‐2. PD‐1 is often coordinately expressed with other co‐inhibitory surface molecules, including LAG‐3, TIM‐3, CD160, BTLA, 2B4, among others.^246^ Together, ligation and activation of the co‐inhibitory receptors can limit TCR‐ and/or co‐stimulatory‐mediated signal propagation. In addition to membrane‐associated inhibitory molecules, a series of intracellular molecules may also serve to downregulate signaling cascades triggered by TCR engagement. For example, proteins involved in the E3‐ligase polyubiquitination pathway, including CBLB^247^ and CISH,^248^ become activated with TCR signaling. Both proteins target key mediators of TCR signaling for proteasomal destruction, limiting T cell‐mediated anti‐tumor responses. PP2A, a serine/threonine phosphatase, negatively regulates AKT‐signaling downstream of TCR ligation in TIL exposed to hyperkalemic tumor interstitial fluid.^249^ Overcoming resistance mediated by negative regulators of TCR signaling is possible. In the case of membrane‐associated co‐inhibitory molecules, infusion of blocking antibodies^17^, ^250^ or genetic engineering with a PD‐1 dominant negative receptor (DNR)^251^ can restore T cell function. For both membrane and intracellular negative regulatory proteins, targeted disruption of genomic loci using CRISPR/Cas9^252^ or other endonucleases^253^ can enhance anti‐tumor immunity.

T cells differentiate in a progressive manner from naive T cells (T_N_) →T stem cell memory (T_SCM_) →T central memory (T_CM_) →T effector memory (T_EM_) and ultimately terminally differentiated effectors (T_EFF_).^23^ Cancer patients frequently are depleted of T_N_ and early memory subsets,^24^, ^25^ which have a superior capacity to proliferate and persist, and develop a reciprocal accumulation of terminally differentiated T_EM_/T_EFF_ cells. These later subsets are prone to apoptosis and incapable of sustained proliferation. Across clinical trials, infusion of cell products lacking T_SCM_/T_CM_ have been associated with impaired T cell expansion and inferior clinical outcomes.^167^, ^254^, ^255^, ^256^ Overcoming acquired T cell‐intrinsic resistance caused by impaired clonal expansion can therefore be accomplished by enriching for minimally‐differentiated T cell subsets prior to TCR engineering^257^ or modifying ex vivo culture conditions to promote T_SCM_/T_CM_ formation.^258^, ^259^ Alternatively, T cells may also be engineered to intrinsically resist apoptosis by disrupting Fas signaling using a DNR approach.^260^

### 5.4 Acquired/late T cell‐extrinsic resistance

Lastly, late/acquired T cell‐extrinsic resistance can result in the loss of an initial clinical response following TCR‐based immunotherapy. This form of resistance is often associated with either loss of function mutations, loss of heterozygosity (LOH), or epigenetic silencing of key genes involved in antigen processing, presentation, and the interferon response pathway. Acquired LOH at the MHC locus was recently reported in two patients receiving ACT of neoantigen‐specific TIL where the specific HLA alleles required for T cell recognition were known.^11^, ^219^ The human genome contains up to six unique HLA class I alleles encoded by three genes, *HLA‐A*, *HLA‐B*, and *HLA‐C*, located in close proximity along the short arm of chromosome 6 (6p).^261^ Given the highly polymorphic nature of the HLA alleles, most individuals are heterozygous for these genes based on the maternal and paternal versions of chromosome 6 they inherit. In both patients, ACT resulted in tumor regression at multiple metastatic sites before a single escape lesion developed. These progressive lesions were surgically resected, permitting detailed immunologic and genomic characterization. The escape lesions continued to express the mutated genes targeted by the infused TIL, excluding antigen loss as the cause of resistance. However, the escape lesions exhibited copy‐neutral LOH at 6p resulting in lost expression of the specific HLA allele required for neoantigen recognition, providing a direct mechanism of immune evasion. Overcoming resistance to HLA LOH can be accomplished by targeting multiple antigens contemporaneously, ideally using TCRs restricted by different parental alleles. Alternatively, promoting tumor antigen cross‐presentation in surrounding stromal cells which retain expression of all HLA molecules can overcome HLA LOH. This can be accomplished by engineering T cells to secrete IL‐12^262^ or other myeloid‐maturation factors.

In addition to LOH, transcriptional repression of individual HLA genes can also mediate acquired resistance to TCR therapies. This was recently demonstrated in two patients who received ACT of CD8^+^ T cells specific for an epitope derived MCPyV.^195^ The MCPyV‐specific T cells in these patients were restricted by HLA‐B*3502 and HLA‐A*02:01, respectively. Following ACT, both patients experienced tumor regression for at ≥18 months before developing progression of disease at a solitary site. Biopsy of the progressing lesion in both patients revealed tumor cell staining with a pan‐HLA class I antibody and an antibody specific for MCPyV. Furthermore, whole exome sequencing failed to detect either mutations or LOH in HLA genes or other genes involved in antigen processing and presentation. These findings suggested that resistance in these cases was not attributable to a complete antigen presentation defect or loss of target antigen expression. However, single cell RNA‐sequencing uncovered a tumor cell‐specific loss of expression for the genes encoding either *HLA‐B* or *HLA‐A*. The loss of expression for a particular HLA gene correlated with the restriction element required for MCPyV recognition in that particular patient. By contrast, non‐transformed cells, such as myeloid, stromal, and endothelial cells, retained expression of both HLA genes. In one patient where tumor cells obtained at the time of progression could be cultured ex vivo, treatment with the hypomethylating agent 5‐azacitadine restored expression of the repressed HLA gene. These findings implicated epigenetic silencing, rather than mutations or chromosomal aberrations, as the pathway of TCR resistance in these patients. Epigenetic modifiers might therefore be used to overcome this pattern of resistance.

## 6 CONCLUDING REMARKS AND OUTLOOK

TCR‐based cancer immunotherapies can mediate durable, and in some cases curative, responses in patients with diverse solid cancers. This finding stands in contrast with current experience using CAR‐based cell therapies outside the context of hematologic malignancies. Although no TCR‐based therapies have yet to receive regulatory approval, it is likely that several will reach this milestone in the coming years. We anticipate that future innovations in TCR‐based therapies will arrive in three epochs, driven by ongoing innovations in genomics, genetic engineering, and cell manufacturing. In the short‐term, current TCR‐based treatments are being combined with adjunctive immunotherapies, including co‐infusion with immune checkpoint inhibitors and immune‐stimulating cytokines. This approach has strong mechanistic rationale as these therapies can overcome multiple pathways associated with TCR resistance. In the next 2‐5 years, new TCR antigen targets will be validated in first in human clinical studies, including additional CGAs, viral oncoproteins, and public neoantigens. Contemporaneously, non‐viral genome editing techniques, including use of CRISPR/Cas9, will test disruption of intracellular and membrane‐associated negative regulatory molecules. Together, the ability to contemporaneously redirect and reprogram the intrinsic functions of T cells will greatly expand the cell engineering tool kit. Finally, in looking beyond the next 5 years, ongoing technologic and manufacturing innovation will make individualized TCR‐based therapies targeting multiple private neoantigens a scalable reality.

## CONFLICT OF INTERESTS

Advisory/consulting: Aleta BioTherapeutics, Bellicum Pharmaceuticals, BMS, Cell Design Labs, G1 Therapeutics, Klus Pharma, Obsidian Therapeutics, Rxi Therapeutics (CAK); Honoraria: Kite/Gilead (CAK); Clinical research support: Kite/Gilead (CAK). CAK and SSC hold provisional patents related to T cell therapy and TCR engineering.
